# Effects of high‐temperature short‐time processing on nutrition quality of Pacific saury (*Cololabis saira*) using extracted fatty acids as the indicator

**DOI:** 10.1002/fsn3.3048

**Published:** 2022-09-17

**Authors:** Kaihui Ding, Yifen Wang, Donglei Luan

**Affiliations:** ^1^ Engineering Research Center of Food Thermal‐Processing Technology Shanghai Ocean University Shanghai China; ^2^ College of Food Science and Technology Shanghai Ocean University Shanghai China; ^3^ Biosystems Engineering Department Auburn University Auburn Alabama USA

**Keywords:** extraction yield, fatty acids, food quality, high‐temperature short‐time (HTST) processing, thermal processing level

## Abstract

Microwave thermal processing is a promising technology to greatly improve product quality by achieving high‐temperature short‐time (HTST) processing for solid foods. And the non‐thermal effect of microwave fields on nutritional quality is a major public concern. To distinguish the non‐thermal effect of microwave fields, the thermal effect of HTST processing should be revealed first. The objective of this study was to investigate the effects of different HTST processing on quality of Pacific saury fillets using extracted fatty acids as the indicator. A self‐developed thermal processing system was used to conduct the HTST processing with different heating rate (5.48–18.30°C/min), maximum heating temperature (123, 133 °C), and thermal processing level (*F*_0_ = 3.0 min, 6.0 min). Results showed that the extraction coefficient of lipids and fatty acids decreased with increasing heating rates, which implied less thermal damage of fish tissue, while higher thermal processing level increased these extraction coefficients. However, higher maximum processing temperature caused serious thermal damage of fatty acids, especially for PUFAs. Furthermore, changing pattern of each fatty acid during different HTST processing was revealed, which provided fundamental data for designing microwave thermal processing and exploring microwave non‐thermal effects.

## Graphical abstract

The HTST processing had great potential to improve the quality of solid foods. However, HTST processing with extra high temperature brought damage to fatty acids.

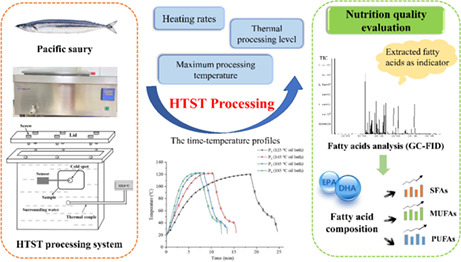

## 1 INTRODUCTION

In food industry, thermal processing is currently the most extensive and effective method for inactivating pathogenic and spoilage microorganisms (Aaliya et al., 2021; Bahrami et al., 2020). In conventional thermal processing, steam or hot water was commonly utilized as the heating medium, and heat transferred from the heating medium to the cold spot area of food through convection and conduction (Bahrami et al., 2020; Guo et al., 2021; Vadivambal & Jayas, 2010). Due to the low thermal conductivity of food materials themselves, it requires a very long time to achieve sufficient thermal lethality of target microbes. However, long‐time thermal processing leads to unavoidable thermal degradation of nutritional and sensory quality for food products (Barbosa‐Cánovas et al., 2014; Cavalcante et al., 2021; Vadivambal & Jayas, 2010).

Generally, thermal processing level (*F*) and cook value (*C*) are used to evaluate the cumulative thermal effect of time and temperature on microorganisms and food quality, respectively. Thermal processing level (*F*) was calculated based on the time‐temperature profile at cold spot to evaluate the thermal lethality of this thermal processing on the objective microbes. It equals a processing time at a reference temperature that brought the same microbial thermal lethality as the conducted thermal processing. The equation for calculating *F* was denoted as:

(1) $$F=\int_{0}^{t}10^{\frac{T\left(t\right)-T_{\mathrm{ref}}}{z}}\mathrm{dt}$$

where *T*(*t*) is the time‐temperature profiles at the cold spot of processed food (°C); *T*_ref_ is the reference temperature; z is the *z*‐value of the target microorganism, which represents its temperature sensitivity; t is the processing time (min). In thermal processing of low‐acid canned foods, *Clostridium botulinum* type A and B (proteolytic) spores are the target bacteria, the corresponding *z*‐value is 10°C. Generally, the reference temperature is chosen to be 121.1°C, while *F* is denoted as *F*_0_ (Tang, 2015). The minimum *F* value for low‐acid canned foods is *F*_0_ = 3.0 min, but *F*_0_ = 6.0 min or longer is usually applied in commercial sterilization (Holdsworth & Simpson, 2016).

Similar to *F*, *C* was used to assess the thermal effect on food quality during thermal processing at the reference temperature. It was calculated according to Equation (2):

(2) $$\begin{array}{c} C=\int_{0}^{t}10^{\frac{T\left(t\right)-T_{\mathrm{ref}}}{z_{n}}}\mathrm{dt} \end{array}$$

where *T*(*t*) is the obtained time‐temperature profiles that same as used in calculating *F*; *T*_ref_ is the reference temperature; z_*n*_ is the *z*‐value for food nutrients, which indicate its sensitivity to the temperature changes. To assess the overall quality loss during thermal processing, the z_*n*_ is usually taken as 33.1°C (Lund, 1986). The reference temperature is chosen to be 100°C, which is designated as *C*_0_.

**FIGURE 1:**
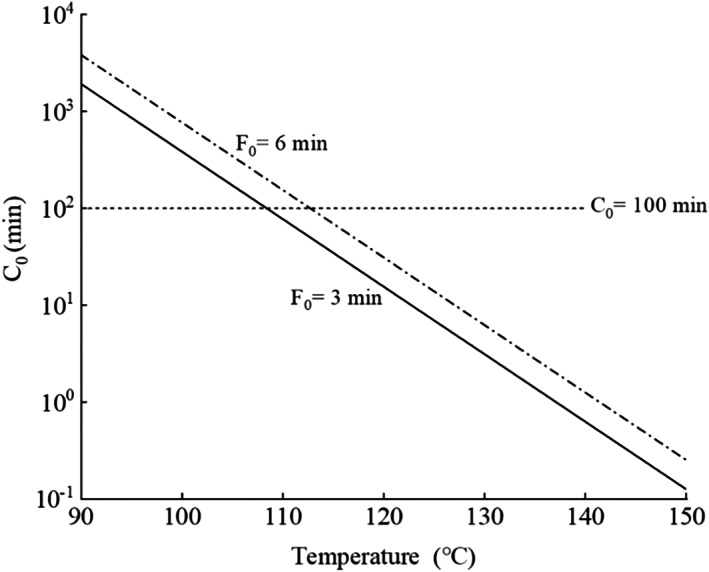
The value of *C*_0_ for different constant temperature

The difference between *z*‐value (7–12°C) for microorganisms and *z*_*n*_‐value (25–45°C) for food nutrients reveals that the thermal resistance of food nutrients is greater than that of microorganisms (Awuah et al., 2007; Holdsworth, 1985; Holdsworth & Simpson, 2016). Generally, for every 10°C rise in temperature, the thermal degradation rate of food nutrients doubles while the microbial lethality rate increases 10‐fold (Awuah et al., 2007). Figure 1 shows the calculated *C*_0_ when the same *F*_0_ value was achieved at different constant temperatures. With the same thermal processing level of *F*_0_ = 3.0 min or 6.0 min, the *C*_0_ value decreased exponentially with increasing processing temperatures. This indicates that thermal processing at higher temperature using a shorter time greatly reduced the thermal degradation of food quality. Theoretically, high‐temperature short‐time (HTST) processing could significantly improve food product quality while ensuring its safety (Awuah et al., 2007; Guo et al., 2021; Peng et al., 2017).

Attribute to the fast heat transfer rate of heat exchanger, the HTST processing method has been successfully achieved in liquids, such as fruit juices, milk, and other drinks (Giribaldi et al., 2016; Matera et al., 2022; Monteiro et al., 2018; Nunes & Tavares, 2019; Pegu & Arya, 2021; Xu et al., 2018). With HTST processing, the nutritional and sensory quality of liquids is greatly improved (Giribaldi et al., 2017; You et al., 2018). Besides, for solid material, only low moisture power (whey proteins and saccharides) was reported, while HTST processing was achieved using high‐temperature oven (130°C) and thinner sample thickness (1 mm) (Liu & Zhong, 2015). In which, HTST processing minimized the undesired color formation and the harmful compounds in the Maillard reaction than conventional processing. Unfortunately, it is very difficult to achieve HTST processing for real solid food products in conventional processing due to their low thermal conductivity. Recently, with the development of novel processing technologies, such as microwave heating and ohmic heating, it becomes practicable to conduct HTST processing for solid food products in near future. However, the effect of HTST processing on quality of real solid foods has not been systematically investigated. Furthermore, microwave thermal processing is the novel technology that has most potential to achieving HTST processing for solid food products. However, besides the well‐known thermal effect, microwave fields also show non‐thermal effect which has been verified on bacteria (Guo et al., 2020, 2021). Then attention of microwave non‐thermal effects was also paid on food nutritional ingredients. The typical characteristic of microwave heating is fast heating rate, that is, HTST processing. Microwave thermal and non‐thermal effect occurs at the same time. Thus, to investigate microwave non‐thermal effect on food nutritional ingredients, it is essential to reveal the thermal effect of HTST processing first.

Fatty acids are essential nutrients that play an important role in numerous functions of our body. Especially, polyunsaturated fatty acids (PUFAs), such as EPA (eicosapentaenoic acid, 20:5n‐3) and DHA (docosahexaenoic acid, 22:6n‐3) could boost immunity and reduce the heart disease risk (Fernandes et al., 2014; Larsen et al., 2010; Matos et al., 2019). However, PUFAs are usually susceptible to oxidation during thermal processing (Gladyshev et al., 2014; Larsen et al., 2010; Zhang et al., 2013). Therefore, fatty acids are one of the most important indicators to evaluate the food product quality, especially for seafood rich in fatty acids such as Pacific saury (*Cololabis saira*).

The objective of this study was to explore the effects of HTST processing on nutrition quality of solid food (Pacific saury fillets). The fatty acids variation was used as the indicator to evaluate the effect of different processing parameters including heating rate (5.48–18.30°C/min), maximum heating temperature (123, 133°C), and thermal processing level (*F*_0_ = 3.0 min, 6.0 min). The obtained results would give new information for the quality control of real solid foods during HTST processing, which could also provide a theoretical basis for the practical application of microwave thermal processing in the future study.

## 2 MATERIALS AND METHODS

### 2.1 Sample preparation

The whole frozen Pacific saury (*Cololabis saira*) was purchased from a local market (Shanghai, China) and stored at −18°C. Before processing, saury was thawed at 0–4°C for 12 h. Fish were headed, gutted and tailed, then cut into three parts along length direction. Then, each part was cut into two fillets along thickness direction with a size of 40 mm × 30 mm × 6 mm for processing and control, respectively. A mobile metallic temperature sensor (PICO VACQ, TMI‐ORION) (Guo et al., 2021) was used to record the time‐temperature profile of saury fillets at the cold spot. Then the fillet with sensor was vacuum packaged in retortable pouches for thermal processing.

### 2.2 Thermal processing design

#### 2.2.1 Thermal processing system

**FIGURE 2:**
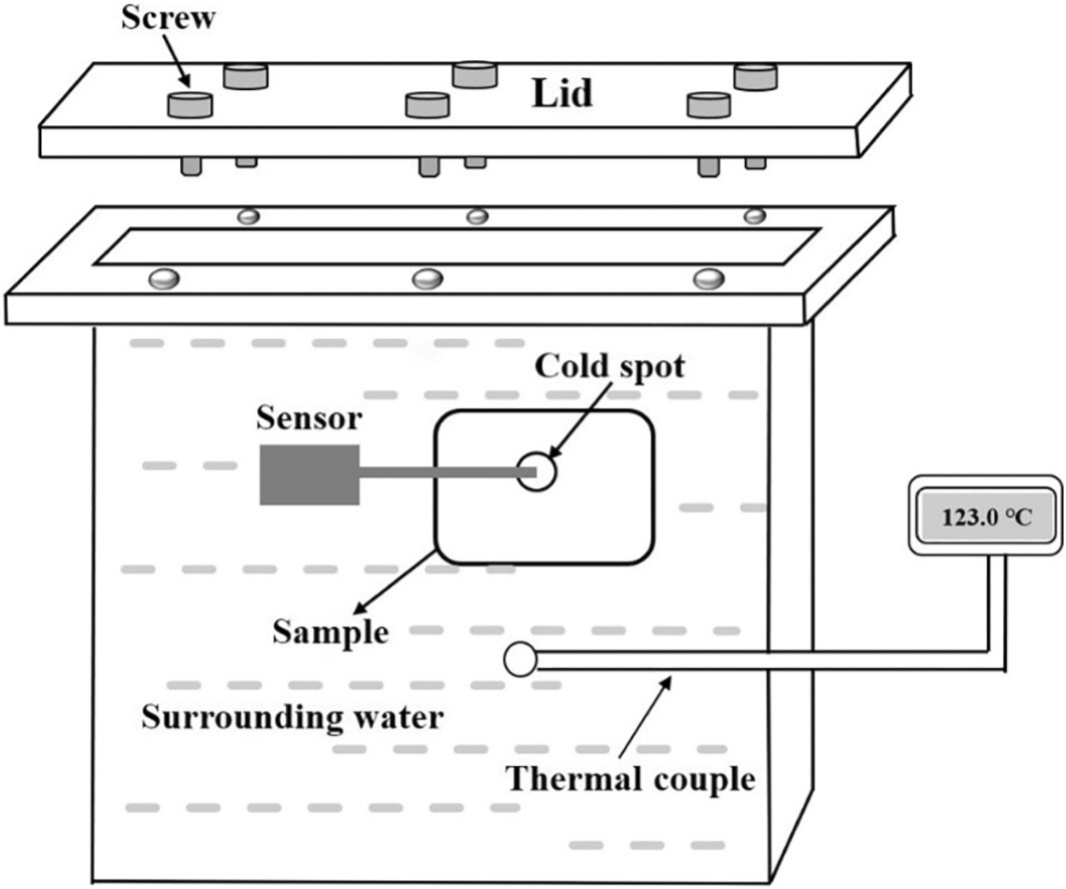
The sketch of the pressure proof container

A thermal processing system was designed to conduct different HTST processing. This system consists of two parts: a heat source and a pressure proof container. An oil bath was used as the heat source for this thermal processing system. Before each experiment, it was preheated to the target temperature. The prepared pressure proof container was then placed into the preheated oil to conduct the thermal processing. The sketch of the pressure proof container is shown in Figure 2. The container was made of aluminum alloy. It consisted of a heating cavity, a matching lid, six screws and a thermal couple. The inner dimension of the heating cavity was 100 mm × 100 mm × 20 mm and the wall thickness were 3 mm.

Before processing, the packaged sample with sensor was placed into the pressure proof container that fulfilled with water (referred as surrounding water). During thermal processing, the surrounding water in the well‐sealed container was heated by the oil bath with different high temperatures. And the food sample was heated by the surrounding water. The real‐time temperature of the surrounding water was monitored by the thermal couple. Once the temperature of surrounding water reached the objective temperature, the container was taken out immediately and then it was placed on top of the hot oil to maintain the temperature, which was called holding. The holding time was determined based on pre‐experiments to obtain the designed *F*_0_. Then the container with the sample was placed into ice water to cool quickly.

#### 2.2.2 Processing with different heating rates

The different heating rates could be realized by varying temperatures of the oil bath. Four different oil bath temperatures, that is, 125, 145, 165, 185°C were adopted to achieve HTST processing with different heating rates. In each experiment, the container with the prepackaged saury fillet was taken out when the temperature of the surrounding water reached 123°C. Then, the same objective thermal processing level (*F*_0_ = 3.0 min) for each processing with different heating rates could be achieved by adjusting the holding time. These four treatments with oil bath temperature of 125, 145, 165, 185°C were recorded as P_1_, P_2_, P_3_, and P_4_, respectively, which were designed to evaluate the effect of different heating rates on the fatty acids of saury fillets.

#### 2.2.3 Processing with different thermal processing levels

Typically, higher thermal processing level leads to more quality loss of processed food. For low‐acid canned food products, the minimum thermal processing level was taken as *F*_0_ = 3 min, but *F*_0_ = 6 min or longer was usually applied in industrial sterilization to highly ensure food safety (Holdsworth & Simpson, 2016). Thus, processing with *F*_0_ = 6.0 min should also take into consideration. With maximum surrounding water temperature of 123°C and target *F*_0_ = 6.0 min, two treatments with different heating rates (125, 185°C oil bath) were designed to investigate the effect of thermal processing levels on fatty acids of saury fillets. These two treatments were recorded as P_5_ and P_6_, respectively.

#### 2.2.4 Processing with different maximum water temperatures

In theoretical, raising the temperature of heating medium is an effective method to shorten the thermal processing time. This is an alternative way to achieve HTST processing. However, extra high temperatures may also cause more serious degradation of food quality, especially for the surface portion. The target temperature for sterilizing low‐acid foods is 121.1°C. In this study of heating rate and thermal processing level, the maximum temperature of heating medium (i.e., surrounding water) was set as 123°C. In order to explore the effect of high temperature of heating medium on the fatty acids of saury fillets, two thermal treatments with maximum water temperature of 133°C were designed. Using the highest heating rate (185°C oil bath), these two treatments with objective thermal processing level of 3.0 and 6.0 min were recorded as processing P_7_ and P_8_, respectively. Each processing was conducted in triplicates.

### 2.3 Analysis of fatty acids

The total lipid was extracted from raw and processed saury based on Bligh and Dyer (1959) method. Then, lipid extracts were transesterified according to the method developed by Metcalfe et al. (1966). The obtained fatty acid methyl esters (FAME) were analyzed with a gas chromatograph TRACE GC ULTRA (Thermo Fisher Inc.), equipped with an Agilent SP‐2560 capillary column (100 m length × 0.25 mm I.D. × 0.2 μm of film) and a flame ionization detector (Thermo Fisher Inc.). The temperature was 260°C for the detector and 250°C for the injector. Nitrogen was used as the mobile phase with a flow rate of 1 ml/min. The injection volume was 1 μl, with a split ratio of 45:1. The temperature program for fatty acid GC analysis was based on the method developed by Zhang et al. (2018): initial temperature was 70°C, increased to 140°C (20°C/min), held for 1 min; then increased to 180°C (4°C/min), held for 1 min; finally increased to 225°C (3°C/min), held for 30 min. Each fatty acid was identified by comparing their retention time with the standard FAME mixtures. The contents of different fatty acids were quantified using the area ratio of peak area between internal standard (C19:0) and different fatty acids.

The extraction yield of lipids and fatty acids could be different with different extraction method and pretreatments (Costa & Bragagnolo, 2017; Gulzar & Benjakul, 2019; Toschi et al., 2003). It was reported that compared with the raw samples, the lipids within thermally processed fatty fish (New Zealand King Salmon) were more effectively extracted using Bligh and Dyer methods. This was attributed to the effects of thermal treatment on the tissue structure and the bound lipids (Larsen et al., 2010). As a result, the extraction yield showed an obvious increase for both lipids and fatty acids. This type of phenomenon was also observed in our pre‐experiments. To clearly describe the effect of different HTST processing on lipids and fatty acids within saury flesh, the extraction coefficient was defined as:

(3) $$\begin{array}{c} \begin{array}{c} \text{Extraction coefficient}\;\left(\%\right)=\frac{\text{Contents of fatty acids or lipids in processed sample}\;}{\text{Contents of fatty acids or lipids in}\;\mathrm{raw}\;\text{sample}\;} \end{array} \end{array}$$

In this study, the calculated extraction coefficient is >100%. Higher extraction coefficient implies more thermal damage to tissue structure and bound lipids. Thus, the changes of the extraction coefficient for lipids and fatty acids at different thermal processing conditions could be an effective indicator to evaluate the quality variation caused by different thermal treatments.

### 2.4 Statistical analysis

Statistical analyses were performed using SPSS statistical software (SPSS 10.0 for Windows; SPSS Inc., Chicago, USA). The independent samples *T* test was performed to evaluate the differences between raw and processed saury samples. Statistical significance was set at *p* < .05.

## 3 RESULTS AND DISCUSSION

### 3.1 Time‐temperature profile, *F*_0_ and *C*_0_

**FIGURE 3:**
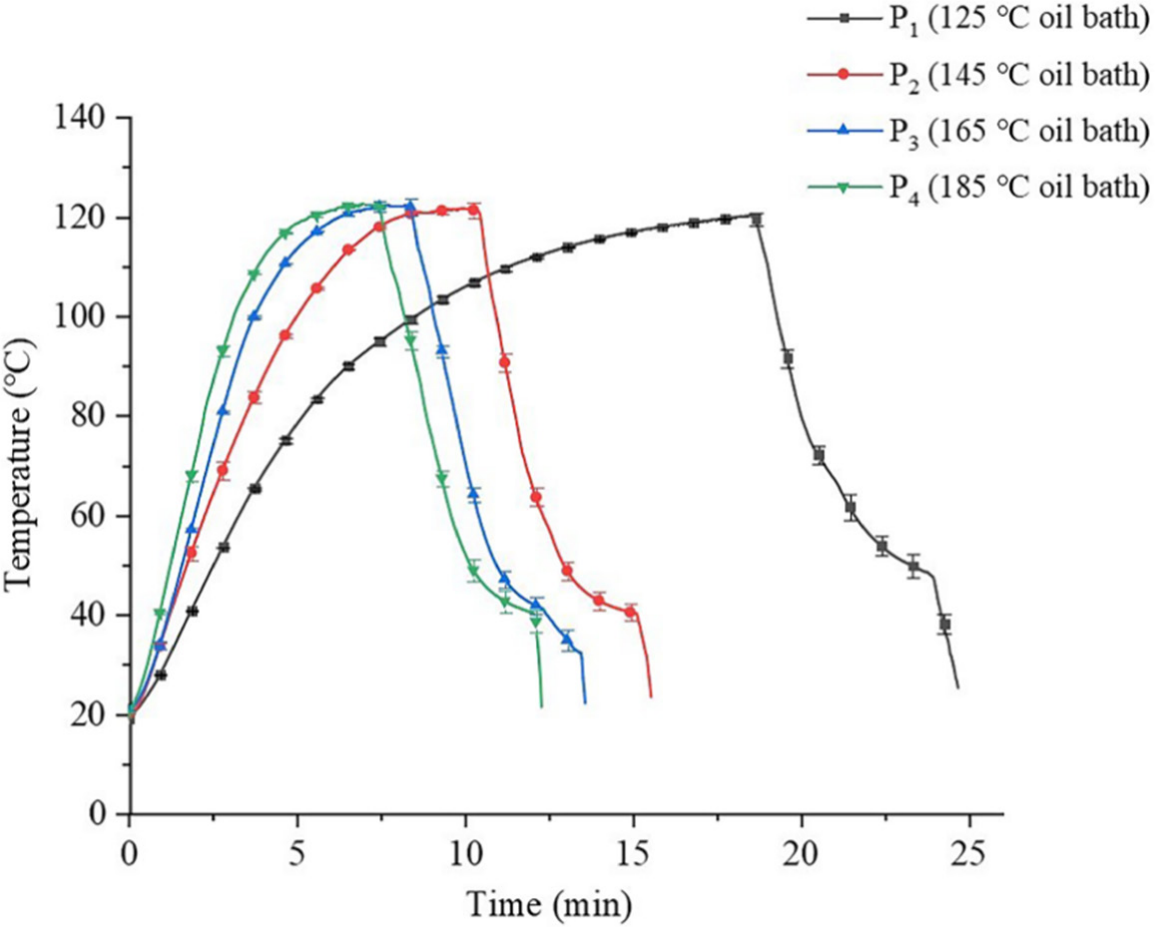
Time‐temperature profile for P_1_, P_2_, P_3_, and P_4_ processing

The time‐temperature profiles of saury fillets processed by HTST processing (P_1_, P_2_, P_3_, and P_4_) are shown in Figure 3. Each curve was plotted based on the average value of triplicates with standard deviations. Results showed that good repeatability was observed for each time‐temperature profile with small standard deviations. This also demonstrated that the designed thermal processing system has good stability for HTST processing of solid foods. In Figure 3, the curvature of each curve gradually decreased with the increasing temperature of oil bath from 125 to 185°C, which showed different heating time and heating rates. In this study, the heating rate for each processing was defined as the temperature increment (from 20 to 120°C) over the time spent for this increment.

The calculated heating rates, *F*_0_ and *C*_0_ for all the conducted processing are shown in Table 1. The results showed that for the processing with the same temperature of oil bath, no significant difference (*p* > .05) was observed for the heating rate. Furthermore, for the processing with the same thermal processing level, there was no significant difference (*p* > .05) in the calculated *F*_0_ values. The results in Table 1 proved that the designed processing was successfully achieved using the thermal processing system.

**TABLE 1:** The parameters for all conducted high‐temperature short‐time (HTST) processing and calculated heating rates, thermal processing levels (*F*_0_) and cook value (*C*_0_)

Processing group	No.	Oil bath temperature (°C)	Maximum water temperature (°C)	Target *F*_0_ (min)	Heating rate (°C/min)	*F*_0_ (min)	*C*_0_ (min)
Different heating rates	P_1_	125	123	3.0	5.48 ± 0.24^d^	3.29 ± 0.07^a^	32.14 ± 0.99^a^
	P_2_	145			12.51 ± 0.37^c^	3.34 ± 0.15^a^	20.55 ± 0.12^b^
	P_3_	165			15.47 ± 0.54^b^	3.31 ± 0.17^a^	18.73 ± 0.18^c^
	P_4_	185			18.30 ± 0.66^a^	3.33 ± 0.23^a^	17.68 ± 0.56^d^
Different thermal processing levels	P_1_	125	123	3.0	5.48 ± 0.24^a^	3.28 ± 0.07^b^	32.14 ± 0.99^b^
	P_5_			6.0	5.63 ± 0.31^a^	6.22 ± 0.10^a^	41.46 ± 1.07^a^
	P_4_	185	123	3.0	18.30 ± 0.66^a^	3.33 ± 0.23^b^	17.68 ± 0.56^b^
	P_6_			6.0	18.56 ± 0.57^a^	6.26 ± 0.26^a^	25.84 ± 0.57^a^
Different maximum water temperatures	P_4_	185	123	3.0	18.30 ± 0.66^a^	3.33 ± 0.23^a^	17.68 ± 0.56^a^
	P_7_		133		18.40 ± 0.76^a^	3.30 ± 0.31^a^	14.64 ± 0.33^b^
	P_6_	185	123	6.0	18.56 ± 0.57^a^	6.26 ± 0.26^a^	25.84 ± 0.57^a^
	P_8_		133		18.67 ± 0.68^a^	6.27 ± 0.37^a^	16.42 ± 0.48^b^

*Note*: Values are mean ± SD of three replicates. The letters in the same column indicate the differences between the corresponding HTST processing groups at a significance level of *p* < .05.

Concerning to cook values with the same *F*_0_ value, it gradually decreased with increasing heating rates. This demonstrated that HTST processing could obviously reduce the quality loss during thermal processing. Generally, higher thermal processing levels result in higher cook values. Compared with low thermal processing level (*F*_0_ = 3.0 min), high level (*F*_0_ = 6.0 min) led to significant increase (*p* < .05) for *C*_0_ from 32.14 to 41.46 min at a low heating rate (125°C oil bath), and from 17.68 to 25.84 min at a high heating rate (185°C oil bath), respectively. With the same increase of *F*_0_ (3 min), the processing with higher heating rate (185°C oil bath) brought lower increment of *C*_0_, which retained more product quality. Furthermore, while the maximum water temperature raised from 123 to 133°C, significant decrease (*p* < .05) on *C*_0_ was observed for thermal processing level of both 3.0 and 6.0 min. This may be attributed to the shorter holding time of the processing with higher maximum water temperature (133°C). These results also showed that HTST processing has a great potential to improve product quality.

### 3.2 Total lipid

The total lipid content of saury fillets during different HTST processing is shown in Table 2. It was difficult to make comparison among different processed saury because of individual differences in raw saury. Thus, the effect of HTST processing on lipid content was investigated by comparing the processed samples with the corresponding raw saury. The total lipid content of processed sample significantly increased (*p* < .05) for all the conducted processing. The extraction coefficient of lipid for each processing is higher than 100%. This indicated that thermal processing improved the lipid extraction of Bligh and Dyer method. It may be due to the thermal damage of these treatments on the fillets, which disrupted the cell structure of saury fillet and facilitated the extraction of lipids (Costa & Bragagnolo, 2017; Gulzar & Benjakul, 2019, 2020; Nieva‐Echevarría et al., 2017). Furthermore, high temperature could also promote the release of binding lipids within the fillets making it easier to extract (Asghari et al., 2013; Larsen et al., 2010; Schneedorferová et al., 2015).

**TABLE 2:** Total lipid content of raw and processed saury during high‐temperatures short‐time (HTST) processing

Processing group	No.	Oil bath temperature (°C)	Target water temperature (°C)	Target *F*_0_ (min)	Total lipid (g/100 g)		Extraction coefficient (%)
					Raw	Processed	
Different heating rates	P_1_	125	123	3.0	11.69 ± 0.48^b^	13.78 ± 0.36^a^	117.85 ± 0.92^a^
	P_2_	145			11.84 ± 0.61^b^	13.56 ± 0.27^a^	114.54 ± 0.84^b^
	P_3_	165			13.17 ± 0.43^b^	14.68 ± 0.41^a^	111.44 ± 0.56c
	P_4_	185			11.94 ± 0.53^b^	13.25 ± 0.45^a^	110.98 ± 0.34^c^
Different thermal processing levels	P_1_	125	123	3.0	11.69 ± 0.48^b^	13.78 ± 0.36^a^	117.85 ± 0.92^b^
	P_5_			6.0	10.66 ± 0.77^b^	12.93 ± 0.62^a^	121.30 ± 0.72^a^
	P_4_	185	123	3.0	11.94 ± 0.53^b^	13.25 ± 0.45^a^	110.98 ± 0.34^b^
	P_6_			6.0	12.18 ± 0.52^b^	13.69 ± 0.27^a^	112.26 ± 0.40^a^
Different maximum water temperatures	P_4_	185	123	3.0	11.94 ± 0.53^b^	13.25 ± 0.45^a^	110.98 ± 0.34^b^
	P_7_		133		12.86 ± 0.56^b^	14.38 ± 0.58^a^	111.81 ± 0.33^a^
	P_6_	185	123	6.0	12.18 ± 0.52^b^	13.69 ± 0.27^a^	112.26 ± 0.40^b^
	P_8_		133		12.10 ± 0.33^b^	13.74 ± 0.60^a^	113.55 ± 0.31^a^

*Note*: Values are mean ± SD of three replicates. Means in the same row for total lipid with different superscripts differ significantly, *p* < .05. The letters in the same column for extraction coefficients indicate differences between the corresponding treatments with a significance level of *p* < .05.

For P_1_, P_2_, P_3_, and P_4_ processing that with same thermal processing level, the extraction coefficient of total lipid gradually reduced from 117.85% to 110.98% while the heating rate increased from 5.48 to 18.30°C/min. These results showed that higher heating rate weakened the extraction coefficient improvement of thermal processing, which implied less thermal damage to the saury fillets. For thermal processing levels, higher *F*_0_ values brought higher extraction coefficient either for processing with high or low heating rate. Furthermore, compared with the maximum water temperature of 123°C, processing with 133°C maximum water temperature produced higher extraction coefficient of total lipid which demonstrated more thermal damage to saury fillets (Gulzar & Benjakul, 2019; Sinthusamran et al., 2018). However, this was different from the results of *C*_0_. This was because that the *C*_0_ was calculated only based on the time‐temperature profiles at cold spot. It cannot represent the whole processed sample. Results of extraction coefficient were more reasonable and higher temperature of heating medium may lead to more thermal damages to processed products.

### 3.3 Fatty acids

The fatty acids of raw and processed samples were analyzed to evaluate the effect of different HTST processing. The extraction coefficient of each fatty acid was calculated to evaluate the variation after HTST processing. These results for all conducted processing are shown in Table 3. The extraction coefficient of total fatty acids (TFAs) for all the processing was higher than 100%, and it was also higher than the extraction coefficient of total lipid (Table 2). This indicated that thermal processing promoted the extraction of fatty acids within saury fillets and this promotion effect was higher than that of the total lipid. It could be attributed to the thermal effect of these treatments on fatty acids, which altered the state of fatty acid in lipids that is, some bound fatty acids were released to free state (Bakar et al., 2008; Duckett & Wagner, 1998; Nieva‐Echevarría et al., 2017; Schneedorferová et al., 2015; Selmi et al., 2008). Thus, more fatty acids were extracted after thermal processing.

**TABLE 3:** The extraction efficiency (%) of fatty acids during different high‐temperature short‐time (HTST) processing

Processing fatty acid	P_1_	P_2_	P_3_	P_4_	P_5_	P_6_	P_7_	P_8_
C12:0	299.23 ± 1.35^b^	217.00 ± 1.30^c^	148.62 ± 0.96^d^	130.79 ± 0.62^f^	328.28 ± 1.12^a^	146.12 ± 0.97^e^	123.67 ± 0.37^h^	124.30 ± 0.75^g^
C13:0	176.82 ± 1.03^b^	171.79 ± 1.27^c^	111.47 ± 0.88^f^	110.97 ± 0.80^g^	182.61 ± 0.96^a^	112.40 ± 0.36^e^	111.28 ± 0.67^f^	113.14 ± 0.25^d^
C14:0	170.60 ± 1.33^b^	155.19 ± 0.61^c^	135.19 ± 0.41^d^	122.03 ± 0.36^f^	180.01 ± 0.61^a^	125.43 ± 0.75^e^	117.28 ± 0.42^g^	122.39 ± 0.31^f^
C15:0	169.75 ± 1.21^b^	136.80 ± 0.91^c^	123.20 ± 0.30^e^	120.76 ± 0.47^f^	181.94 ± 1.07^a^	126.02 ± 0.37^d^	120.30 ± 0.76^f^	120.21 ± 0.85^f^
C16:0	147.40 ± 0.95^b^	133.84 ± 0.89^c^	125.04 ± 0.96^d^	122.07 ± 0.14^f^	179.82 ± 0.98^a^	123.50 ± 0.45^e^	119.80 ± 0.26^g^	122.35 ± 0.64^f^
C17:0	135.21 ± 0.86^b^	126.37 ± 0.82^c^	121.16 ± 0.23^e^	115.08 ± 0.56^f^	148.25 ± 1.01^a^	122.47 ± 0.69^d^	115.23 ± 0.58^f^	121.22 ± 0.51^e^
C18:0	130.76 ± 0.76^b^	127.71 ± 0.42^c^	124.11 ± 0.60^d^	115.80 ± 0.41^f^	136.97 ± 0.42^a^	120.59 ± 0.37^e^	115.17 ± 0.82^f^	120.15 ± 0.26^e^
C20:0	134.72 ± 0.63^b^	124.07 ± 0.73^c^	121.60 ± 0.86^e^	121.06 ± 0.95^e^	141.51 ± 0.91^a^	122.04 ± 0.72^d^	111.28 ± 0.37^f^	102.57 ± 0.61^g^
C21:0	124.08 ± 0.38^c^	122.91 ± 0.29^d^	113.79 ± 0.35^g^	112.02 ± 0.70^h^	145.55 ± 0.43^a^	128.60 ± 0.30^b^	118.82 ± 0.29^f^	121.92 ± 0.29^e^
∑SFAs	152.70 ± 1.07^b^	139.99 ± 0.32^c^	127.85 ± 0.32^d^	121.13 ± 0.96^f^	160.28 ± 0.73^a^	124.23 ± 0.13^e^	111.28 ± 0.66^h^	113.14 ± 0.74^g^
C14:1	235.76 ± 1.30^b^	152.70 ± 0.51^c^	148.62 ± 0.22^d^	138.71 ± 0.70^f^	252.16 ± 1.13^a^	148.33 ± 0.51^d^	134.37 ± 0.67^g^	141.43 ± 0.68^e^
C16:1	176.16 ± 0.42^b^	153.33 ± 0.67^c^	135.11 ± 0.87^d^	114.67 ± 0.41^g^	194.77 ± 0.53^a^	118.96 ± 0.19^e^	114.18 ± 0.40^g^	117.41 ± 0.62^f^
C18:1n‐9 t	187.35 ± 1.31^b^	160.34 ± 0.98^c^	130.04 ± 0.91^e^	126.82 ± 0.97^f^	189.94 ± 0.31^a^	134.51 ± 0.67^d^	122.17 ± 0.97^h^	125.71 ± 0.40^g^
C18:1n‐9 c	184.15 ± 0.99^b^	150.85 ± 0.89^c^	128.42 ± 0.35^d^	119.59 ± 0.34^f^	230.33 ± 1.06^a^	120.89 ± 0.55^e^	118.10 ± 0.53^g^	120.57 ± 0.49^e^
C20:1	167.73 ± 0.66^b^	136.73 ± 0.90^c^	126.79 ± 0.95^d^	121.83 ± 0.57^f^	179.73 ± 0.34^a^	126.64 ± 0.29^d^	119.53 ± 0.85^g^	125.66 ± 0.29^e^
C22:1n‐9	144.77 ± 1.00^b^	129.22 ± 1.13^c^	122.19 ± 0.10^f^	121.97 ± 0.46^f^	145.77 ± 0.90^a^	126.87 ± 0.60^d^	120.41 ± 0.74^g^	123.65 ± 0.17^e^
C24:1	162.41 ± 0.89^b^	138.31 ± 0.74^c^	125.84 ± 0.20^d^	118.90 ± 0.48^f^	180.53 ± 0.53^a^	124.11 ± 0.85^e^	118.71 ± 0.53^f^	124.02 ± 0.25^e^
∑MUFAs	160.19 ± 0.82^b^	134.26 ± 0.52^c^	125.96 ± 0.33^d^	121.56 ± 0.29^f^	169.39 ± 0.81^a^	125.52 ± 0.27^d^	119.59 ± 0.27^g^	124.11 ± 0.82^e^
C18:2n‐6 t	242.04 ± 1.21^b^	143.58 ± 0.75^d^	138.00 ± 0.92^e^	117.60 ± 0.63^g^	288.40 ± 1.24^a^	148.65 ± 0.68^c^	114.84 ± 0.20^h^	134.28 ± 0.94^f^
C18:2n‐6 c	201.82 ± 0.63^b^	147.61 ± 0.64^c^	132.81 ± 0.88^f^	131.03 ± 0.97^g^	212.68 ± 0.97^a^	143.87 ± 0.30^d^	128.21 ± 0.71^h^	136.40 ± 0.64^e^
C18:3n‐3	220.04 ± 0.96^b^	157.74 ± 0.15^c^	139.99 ± 0.33^f^	134.62 ± 0.55^g^	237.88 ± 1.02^a^	156.21 ± 0.57^d^	132.65 ± 0.84^h^	145.71 ± 0.53^e^
C18:3n‐6	177.17 ± 0.75^b^	148.89 ± 0.54^d^	130.04 ± 0.30^f^	128.71 ± 0.67^g^	197.10 ± 0.30^a^	163.71 ± 0.86^c^	123.82 ± 0.23^h^	145.47 ± 0.64^e^
C20:2n‐6	217.10 ± 1.27^b^	177.69 ± 0.72^c^	145.37 ± 0.27^e^	141.13 ± 0.83^g^	239.05 ± 0.78^a^	159.79 ± 0.91^d^	132.20 ± 0.26^h^	143.96 ± 0.83^f^
C20:3n‐6	147.35 ± 0.77^a^	114.53 ± 0.88^c^	111.47 ± 0.32^e^	110.97 ± 0.66^e^	141.51 ± 0.86^b^	113.50 ± 0.32^d^	111.28 ± 0.61^e^	113.14 ± 0.56^d^
C20:3n‐3	241.45 ± 1.07^b^	179.97 ± 0.85^c^	117.66 ± 0.40^f^	114.93 ± 0.91^g^	269.92 ± 0.81^a^	121.13 ± 0.86^e^	110.22 ± 0.26^h^	124.45 ± 0.48^d^
C20:4n‐6 (ARA)	186.12 ± 0.46^b^	158.16 ± 0.73^c^	134.69 ± 0.44^e^	127.41 ± 0.90^g^	205.13 ± 0.90^a^	139.37 ± 0.72^d^	120.51 ± 0.67^h^	130.55 ± 0.42^f^
C20:5n‐3 (EPA)	193.82 ± 0.79^b^	161.86 ± 0.85^c^	144.12 ± 0.36^d^	140.44 ± 0.23^f^	260.85 ± 1.18^a^	161.38 ± 0.36^c^	136.49 ± 0.74^g^	145.72 ± 0.15^e^
C22:2n‐6	218.42 ± 1.07^b^	173.12 ± 1.01^c^	143.73 ± 0.91^e^	134.75 ± 0.38^g^	247.71 ± 1.04^a^	149.86 ± 0.38^d^	128.07 ± 0.68^h^	135.36 ± 0.41^f^
C22:6n‐3 (DHA)	147.93 ± 0.82^b^	133.64 ± 0.5^1^c	121.42 ± 0.78^f^	115.38 ± 0.49^g^	172.29 ± 0.66^a^	124.98 ± 0.24^d^	113.92 ± 0.37^h^	122.66 ± 0.22^e^
∑PUFAs	178.75 ± 0.66^b^	147.99 ± 0.96^c^	132.32 ± 0.79^f^	125.76 ± 0.28^g^	211.19 ± 0.75^a^	138.67 ± 0.51^d^	123.18 ± 0.39^h^	133.86 ± 0.71^e^
TFAs	164.45 ± 0.69^b^	140.15 ± 0.48^c^	128.53 ± 0.28^e^	122.90 ± 0.82^g^	177.86 ± 0.86^a^	129.55 ± 0.35^d^	120.63 ± 0.31^h^	126.64 ± 0.68^f^

*Note*: Values are mean ± SD of three replicates. Different lowercase letters within the same row indicate significant difference (*p* < .05).

Abbreviations: ∑SFAs, total saturated fatty acids; ∑MUFAs, total monounsaturated fatty acids; ∑PUFAs, total polyunsaturated fatty acids; EPA, eicosapentaenoic acid; DHA, docosahexaenoic acid; TFAs, total fatty acids.

#### 3.3.1 Effects of heating rate on fatty acids

The processing P_1_, P_2_, P_3_, and P_4_ were designed to investigate the effect of heating rate on fatty acids. The extraction coefficient of TFAs were 164.45%, 140.15%, 128.53%, and 122.90% for P_1_, P_2_, P_3_, and P_4_ processing, respectively (Table 3). The results showed that with same thermal processing level (*F*_0_ = 3.0 min), the extraction coefficient of TFAs gradually reduced while the heating rate increased from 5.48 to 18.30°C/min. For the three fatty acids categories of saturated fatty acids (SFAs), monounsaturated fatty acids (MUFAs) and PUFAs, same trend was observed. Results demonstrated that higher heating rate reduced the effect of thermal processing on the fatty acids within saury fillets.

Concerning to each fatty acid, there were 18, 16, 13, and 10 kinds of fatty acids that showed higher extraction coefficient than TFAs for P_1_, P_2_, P_3_, and P_4_ processing, respectively (Table 3). Extraction of these fatty acids was more sensitive to thermal processing than the others. Furthermore, most of them were PUFAs. As a result, different from SFAs and MUFAs, the PUFAs showed a higher extraction coefficient than TFAs at each corresponding heating rate. This is similar to previous report that: the state of PUFAs within fish fillets was more sensitive to thermal processing (Nieva‐Echevarría et al., 2018, 2017; Selmi et al., 2008).

#### 3.3.2 Effects of thermal processing level on fatty acids

The effect of different thermal processing levels (*F*_0_ = 3.0 and 6.0 min) on fatty acids was also investigated with the maximum water temperature of 123°C and different heating rates of 5.48°C/min (P_5_) and 18.30°C/min (P_6_).

With the heating rate of 5.48°C/min, the extraction coefficient of TFAs and PUFAs increased by 13.41 (from 164.45% to 177.86%) and 32.44 (from 178.75% to 211.19%) percentage points, respectively, when the thermal processing level increased from 3.0 (P_1_) to 6.0 min (P_5_). While with heating rate of 18.30°C/min, the extraction coefficient of TFAs and PUFAs increased by 6.65 (from 122.90% to 129.55%) and 12.91 (from 125.76% to 138.67%) percentage points, respectively, when the thermal processing level extended from 3.0 (P_4_) to 6.0 min (P_6_). Results showed that the higher thermal processing level obviously increased the extraction coefficient of TAFs and PUFAs for processing with high or low heating rate. This indicated that higher thermal processing levels brought more thermal effect to the state of fatty acids within saury fillet. Moreover, with higher thermal processing levels, the number of fatty acids that showed higher extraction coefficients than TFAs increased by one and two for P_5_ and P_6_ processing, respectively.

Furthermore, at higher heating rate of 18.30°C/min, smaller increments for extraction coefficient were observed for TFAs and PUFAs, which agreed with the results of heating rate effect. This indicated that high heating rate could reduce the thermal effect caused by higher thermal processing level on fatty acids state within saury fillets.

#### 3.3.3 Effects of maximum water temperature on fatty acids

Raising heating medium temperature is an alternative method to increase heating rate in traditional thermal processing. Two thermal treatments with maximum water temperature of 133°C were conducted to explore the effect of high heating medium temperature on the fatty acids in saury fillets. Using the highest heating rate treatment (185°C oil bath), the objective thermal processing level of these two treatments were 3.0 (P_7_) and 6.0 min (P_8_), respectively.

The extraction coefficient results (Table 3) of fatty acids showed that with thermal processing level of *F*_0_ = 3.0 min, the extraction coefficient of TFAs and PUFAs decreased by 2.27 (from 122.90% to 120.63%) and 2.58 (from 125.76% to 123.18%) percentage points, respectively, when the maximum water temperature increased from 123°C (P_4_) to 133°C (P_7_). Furthermore, with a higher thermal processing level of *F*_0_ = 6.0 min, the decrease for the extraction coefficient of TFAs and PUFAs were 2.91(from 129.55% to 126.64%) and 4.81 (from 138.67% to 133.86%) percentage points, respectively, while maximum water temperature increased from 123°C (P_6_) to 133°C (P_8_). These results demonstrated that high temperature of heating medium reduced the extraction coefficient of TFAs and PUFAs, especially at higher thermal processing level. Furthermore, with higher maximum water temperature, the number of fatty acids that showed higher extraction coefficients than TFAs decreased by one and two for P_7_ and P_8_ processing, respectively.

This was not consistent with the effect of higher water temperature on lipids. With higher heating medium temperature of 133°C, the extraction coefficient of lipids increased by 0.83 (from 110.98% to 111.81%) and 1.29 (from 112.26% to 113.55%) percentage points, respectively, for *F*_0_ = 3.0 and 6.0 min. In theoretical, higher temperature and higher thermal processing level brought more intensive effect on lipids and fatty acids within saury fillets. The reduction of TFAs and PUFAs were attributed to the explanation that the higher temperature aggravated the oxidation of fatty acids, especially PUFAs (Amaral et al., 2018; Domínguez et al., 2019; Min & Ahn, 2005; Roldan et al., 2014). This was a reasonable explanation while the reduction of PUFAs (2.58 and 4.81 percentage points) was more compared with TFAs (2.27 and 2.91 percentage points). Because PUFAs were more susceptible to oxidation at high temperature (Amira et al., 2010; Zhang et al., 2013). As a result, extra high temperature of heating medium was not a good method to achieve HTST processing while it brought damage to PUFAs. For microwave thermal processing, the temperature at hot spot should be controlled to maintain product quality.

## 4 CONCLUSION

In this study, a self‐designed pressure proof container was used to achieve the high‐temperature short‐time (HTST) processing of solid food products. The effects of different HTST processing on solid food quality were investigated by analyzing the extraction coefficient of fatty acids in Pacific saury fillets. Results showed that the total lipid content significantly increased (*p* < .05) in all processed samples compared with raw samples. This indicated that thermal processing could obviously alter the lipid state within the saury fillets and improve extraction yield.

With increasing heating rates, the extraction coefficient of total fatty acids (TFAs) and polyunsaturated fatty acids (PUFAs) gradually reduced. However, at each heating rate, the extraction coefficient of PUFAs was higher than that of TFAs, which indicated that the state of PUFAs within saury was more sensitive to thermal treatments. Results demonstrated that higher heating rate brought less thermal effect on fatty acids within saury fillets. This proved the potential of HTST processing in reducing quality degradation during thermal processing.

With the thermal processing level increasing from 3.0 to 6.0 min, the extraction coefficient of TFAs and PUFAs increased by 13.41 and 32.44 percentage points at the heating rate of 5.48°C/min, and 6.65 and 12.91 percentage points at heating rate of 18.30°C/min, respectively. This revealed that higher thermal processing level led to more thermal effect to the fatty acids within saury fillet. However, with maximum surrounding water temperature increasing from 123 to 133°C, the extraction coefficient of both TFAs and PUFAs decreased, especially at higher thermal processing level of *F*_0_ = 6.0 min. This was attributed to the oxidation of fatty acids, particularly PUFAs, at high temperature. Thus, raising the heating medium temperature was not a suitable way to achieve HTST processing and obtain good product quality.

The HTST processing is a good methodology to retain the quality of solid food products during thermal processing, which verified the advantages of microwave thermal processing. In order to keep its advantages, the temperature at hot spot of microwave processing should be controlled. Furthermore, the changing pattern of each fatty acid after different HTST processing were systematically revealed. These results provided essential data for exploring microwave non‐thermal effects.

## CONFLICT OF INTEREST

We declare that we have no financial and personal relationships with other people or organizations that can inappropriately influence our work.

## ETHICAL APPROVAL

Ethics approval was not required for this research.

## Data Availability

The data that support the findings of this study are available from the corresponding author upon reasonable request.
